# Real‐world analysis of the relationships between smoking, lung cancer stigma, and emotional functioning

**DOI:** 10.1002/cam4.6702

**Published:** 2024-01-12

**Authors:** Kari Chansky, Maureen Rigney, Jennifer C. King

**Affiliations:** ^1^ Chansky Consulting LLC Mercer Island Mercer Island Washington USA; ^2^ Fred Hutchinson Cancer Center Seattle Washington USA; ^3^ GO2 for Lung Cancer Washington DC USA

**Keywords:** lung neoplasms, patient reported outcome measures, quality of life, smoking, stigmatization

## Abstract

**Introduction:**

People diagnosed with lung cancer experience high rates of distress, which can be compounded by the stigma of the disease. This study assessed a real‐world population to understand patient‐reported emotional functioning, types of stigma experienced, and relationship with smoking history.

**Methods:**

Questionnaires using validated survey tools assessing demographics, smoking history, stigma, and quality of life (EORTC QLQ‐C30 Emotional Functioning Scale) were analyzed from 539 global participants in the Lung Cancer Registry between November 2019 and July 2022. The associations between smoking history and self‐reported internalized and perceived stigma and constrained disclosure of lung cancer diagnosis, as well as the potential impact of stigma on emotional functioning, were examined using multivariable logistic regression models.

**Results:**

Among the broad geographic mix of study participants, all types of lung cancer stigma were associated with decreased emotional functioning due to a combination of factors including depression, anxiety, stress, and irritability. Participants who reported a history of current or former smoking experienced higher levels of internalized stigma and perceived stigma. Constrained disclosure about a diagnosis was common, associated with decreased emotional functioning, and not related to a history of smoking. Smoking status itself was not associated with reduced emotional functioning, implicating the role of stigma in distress.

**Conclusions:**

In this study, all types of lung cancer stigma were associated with clinically important decreases in emotional functioning. This impact was not dependent on smoking history. Internalized and perceived stigma were associated with the presence of a smoking history. These findings have implications for proper psychosocial care of people diagnosed with lung cancer.

## 1 INTRODUCTION

Lung cancer is the leading cause of cancer death in the United States and worldwide.^1^, ^2^ Globally, it is the leading cause of cancer death for men and second only to breast cancer for women, while in the United States, it is the leading cause for both sexes. Each year in the United States, over 230,000 people are diagnosed with lung cancer, and more than 130,000 will die from the disease.^3^ Often found only after it has spread, the overall 5‐year survival rate of lung cancer is only 22%.^3^ As a disease with high symptom and treatment side effect burden,^4^, ^5^ research has shown that people diagnosed with lung cancer report higher levels of distress^6^ compared with other types of cancer and experience great unmet physical and emotional needs.^7^

One important component of this distress is the presence of lung cancer stigma. Although rates vary, one study found that 95% of people diagnosed with lung cancer had experienced stigma in one form or another.^8^ Despite significant improvements in the treatment paradigm for lung cancer, previous research has shown that over a 10‐year period, people diagnosed perceived an increase in stigma associated with lung cancer and that those diagnosed are treated differently.^9^

The examination of behavior and condition‐related stigma began in 1963 with Erving Goffman's seminal work, Stigma: Notes on the Management of Spoiled Identity.^10^ Per Goffman, the word stigma originated with the Greeks, and referred to “bodily signs designed to expose something unusual or bad about the moral status of the signifier.” These were inflicted onto the body to indicate the individual as “a slave, a criminal, or a traitor—a blemished person, ritually polluted, to be avoided, especially in public places.” The concept of stigma has evolved over time, as Goffman recognized, saying it is now “something like the original literal sense, but is applied more to the disgrace itself than to the bodily evidence of it.”

Goffman's book was published just prior to release of the Surgeon General's 1964 Report on Smoking and Health, a time when smoking cigarettes and people with lung cancer were not yet stigmatized.^11^ Forty years later, in 2003, Kim outlined how the recognition of the health risks of smoking cigarettes led to well‐meaning attempts to prevent smoking initiation and encourage cessation.^12^ Kim also recognized that after the links between secondhand smoke and health effects became known in the early 1990's, cigarette use moved from a personal to a social problem, and the stigmatization of people who smoke began. As anti‐smoking bans were enacted and other tobacco control efforts came to prominence, people who smoked were ostracized, “rejected from full social acceptance,” and regarded as “deviants.”

In 2004, Chapple published the first study to link lung cancer and stigma as a result of the disease's association with smoking.^13^ Chapple categorizes the stigma around lung cancer through the lens of other stigmatized diseases, of being “felt” or “enacted.” Shame and fear of reprisal one may feel were identified as felt stigma, with enacted stigma being identified discrimination as a result of the diagnosis.

Since then, research has revealed the detrimental societal, interpersonal, and personal impacts of lung cancer stigma. Those with a history of smoking who are diagnosed may feel shame and guilt.^14^, ^15^, ^16^ Anyone, regardless of smoking history, can be subjected to stigmatizing questions and comments by strangers and acquaintances^16^, ^17^ Loved ones may say or do things that are stigmatizing,^18^, ^19^ and differential treatment by medical professionals may be delivered or perceived.^20^, ^21^

Stigma has been shown to have significant effects on emotional functioning and quality of life.^22^, ^23^ One out of four people with lung cancer experience periods of depression or other psychosocial problems.^24^ People affected by lung cancer stigma have been found to have increased rates of depression^25^, ^26^ and isolation^27^, ^28^ In 2012, Cataldo found that, regardless of smoking history, lung cancer stigma was correlated with high rates of depression and lower quality of life^23^ Additionally, when and how people with lung cancer seek treatment may be affected.^15^, ^29^

A scoping review by Webb et al.^22^ outlines the variety of ways that the levels of stigma have evolved and changed since Chapple. Different ways to measure lung cancer stigma and a variety of ways of describing the types and levels have made cross‐study comparison difficult. In 2018, Hamann et al.^30^ conceptualized lung cancer stigma as “a form of health‐related stigma in which a person perceives and potentially internalizes an experience of rejection, blame, or devaluation directly linked to the belief that their behavior (i.e., smoking) has caused their current health condition (i.e., lung cancer).” They employed a multi‐phase process to develop the multi‐dimensional measure of lung cancer stigma with the Lung Cancer Stigma Inventory (LCSI).^30^ The LCSI provides a common language and standard assessment to measure how stigma is experienced and identifies three “unique, internally consistent, and conceptually relevant factors” relevant to the person diagnosed:

- Perceived stigma, the negative appraisal and devaluation from others.
- Internalized stigma, self‐blame, guilt, and regret.
- Constrained disclosure, if and how people share their diagnosis, may result in social avoidance and disengagement.

The direct relationship between smoking status and lung cancer stigma is a relatively unexplored area of focus. Williamson's 2018 small, site‐based study using the LCSI found that internalized stigma but not constrained disclosure was correlated with smoking history.^31^ It is unknown how smoking status and stigma may relate in the more generalizable setting of individuals with lung cancer accessing routine care. Real‐world data are data that relate to patient health status and/or the delivery of health care and are routinely collected from a variety of sources.^32^ The present study utilizes a large, real‐world data set from a disease registry to expand upon findings concerning the associations between smoking and stigma and to characterize the impact of stigma on the emotional functioning of lung cancer patients.

## 2 METHODS

### 2.1 Platform & questionnaire

The study sample was selected from all consented participants in the Lung Cancer Registry^33^, ^34^ (www.lungcancerregistry.org) based upon availability of complete data for smoking history and responses to questions about internalized stigma, perceived stigma, and constrained disclosure. The data cutoff was July 15, 2022. Since 2016, the Lung Cancer Registry has been collecting information directly from patients and caregivers about their experience with lung cancer across the continuum of diagnosis and care. Participants are asked to take a baseline survey upon enrollment and then prompted to complete a longitudinal update survey every 3 months. As of October 2023, there have been 2584 total participants. In November 2019, the longitudinal core surveys were expanded, IRB approved, and the smoking history questions were updated to align with nationally recognized definitions.^35^ For this reason, responses were only taken from survey submissions that occurred after this revision. Consequently, the dataset contains complete responses between November 21, 2019 and July 15, 2022.

The baseline and longitudinal surveys are comprehensive and cover many aspects of diagnosis, treatment, and survivorship. Stigma questions were taken from the Lung Cancer Stigma Inventory.^30^ The most highly correlated question for each type of stigma subscale (internalized stigma, perceived stigma, and constrained disclosure) with the highest factor loading was added to the registry questionnaire during the 2019 revision. Stigma was reported as a 5‐level scale ranging from “Not at all” (no stigma felt) to “Extremely.” This multilevel response was explored with respect to smoking history, and for simplicity, the final modeling used a binary indicator of stigma (“Not at all” versus any other level of stigma) as the outcome. Smoking history questions were core questions 1, 4, 5, and 6 from the NCI Cancer Patient Tobacco Use Questionnaire (C‐TUQ).^36^ Quality of Life assessments within the longitudinal survey include the full EORTC QLQ‐C30 (v3.0),^37^, ^38^ a widely used questionnaire developed by the EORTC to assess the quality of life of cancer patients.

### 2.2 Analysis

For each subject, the first record with a response related to stigma was selected, and all responses related to stigma and emotional functioning were drawn from that time point. Smoking history was taken from the first survey where the appropriate smoking history questions were administered, and the smoking status as defined below was determined from that time point. The analysis therefore focuses on one set of responses per qualifying subject, where responses can be from time points other than the initial survey submission. Time from initial participation in the survey, which is a potential proxy for time from initial lung cancer diagnosis and could have an effect on feelings of stigma, was therefore included as a covariate.

Smoking history was dichotomized to classify participants as no smoking history (less than 100 cigarettes smoked in one's lifetime) versus smoking history (including current or former smoking). This dichotomy reflects the perceived impact of a history of smoking on an individual's lung cancer risk, regardless of when the individual quit. Age was considered as a potential factor and modeled as the current age at the time of survey submission and dichotomized for simplicity of interpretation to <70 versus 70 and older. This age cutpoint was chosen based on the timing of the 1964 Surgeon General Report^11^ on the risks of smoking and patterns of changing societal smoking habits.

Participant age, time from initial survey submission, sex, and geographic region were all explored in univariable models for an association with stigma. Covariates that were significant were included in the final multivariable models with smoking status. Individual multivariable logistic regression models for each type of stigma, with the binary indicator of the reporting of any level of stigma as the response, were performed. Within participants who did not have a history of smoking, cell sizes for individual levels of stigma on the 5‐level scale were generally too small for a valid analysis of stigma as an ordered response. Ordered logistic regression models were therefore performed only as sensitivity analyses, with standard logistic regression on the binary variables serving as the primary analysis method. Tests for interactions between the smoking history and other covariates were done to rule out potential effect modifications.

The Emotional Functioning Scale score from the EORTC QLQ‐C30^37^ was used to examine impacts of stigma on the person with lung cancer. Scores were examined both as a continuous variable in association with stigma as well as dichotomized using a previously developed threshold for clinical importance (TCI) of 71 points on a 0–100 scale.^39^

## 3 RESULTS

There were 539 complete cases from participants with data on stigma, constrained disclosure, smoking history, emotional functioning score, and other demographic variables including age (Table 1). There was broad geographic representation across all regions of the United States, and 11.8% of participants were international. Seventeen countries outside of the United States were represented.

**TABLE 1:** Demographics of the real‐world sample from the registry.

Patient Characteristics	Ever Smoked				All
	No		Yes		
All Participants	267	50%	272	50%	539
Age at survey submission					
Under 40	24	86%	4	14%	28
40 to <50	42	67%	21	33%	63
50 to <60	91	60%	61	40%	152
60 to <70	76	41%	109	59%	185
70 to <80	31	32%	67	68%	98
80+	3	23%	10	77%	13
Gender					
Female	216	51%	207	49%	423
Male	51	44%	65	56%	116
Race					
Asian	24	100%	0	0	24
Black or Afr. Amer.	4	50%	4	50%	8
Multiracial/other	0	0	1	100%	1
White	222	47%	253	53%	475
Missing	17	55%	14	45%	31
Type/stage					
NSCLC‐stage I	31	39%	49	61%	80
NSCLC‐stage II	11	34%	21	66%	32
NSCLC‐stage III	43	43%	56	57%	99
NSCLC‐stage IV	164	62%	102	38%	266
NSCLC‐stage unsure	11	37%	19	63%	30
SCLC	7	22%	25	78%	32
Region					
International	40	63%	24	38%	64
Midwest	52	50%	52	50%	104
Mountain	14	58%	10	42%	24
Northeast	44	47%	50	53%	94
Pacific	62	53%	54	47%	116
South	55	40%	82	60%	137

Fifty percent of participants identified as having a smoking history (>100 cigarettes in their lifetime), and smoking history was balanced across male and female gender. Older participants were more likely to have a smoking history than younger participants (69% 70 and older vs 44% under 70). Participants who presented with stage IV non‐small cell lung cancer were less likely have a history of smoking (39% vs 63% with other stages). Twenty‐five of the 32 participants reporting limited or extensive stage SCLC report a history of smoking (78%), a higher rate than in the remaining subjects of 49% (*p* = 0.0016, Fisher's exact test).

**FIGURE 1:**
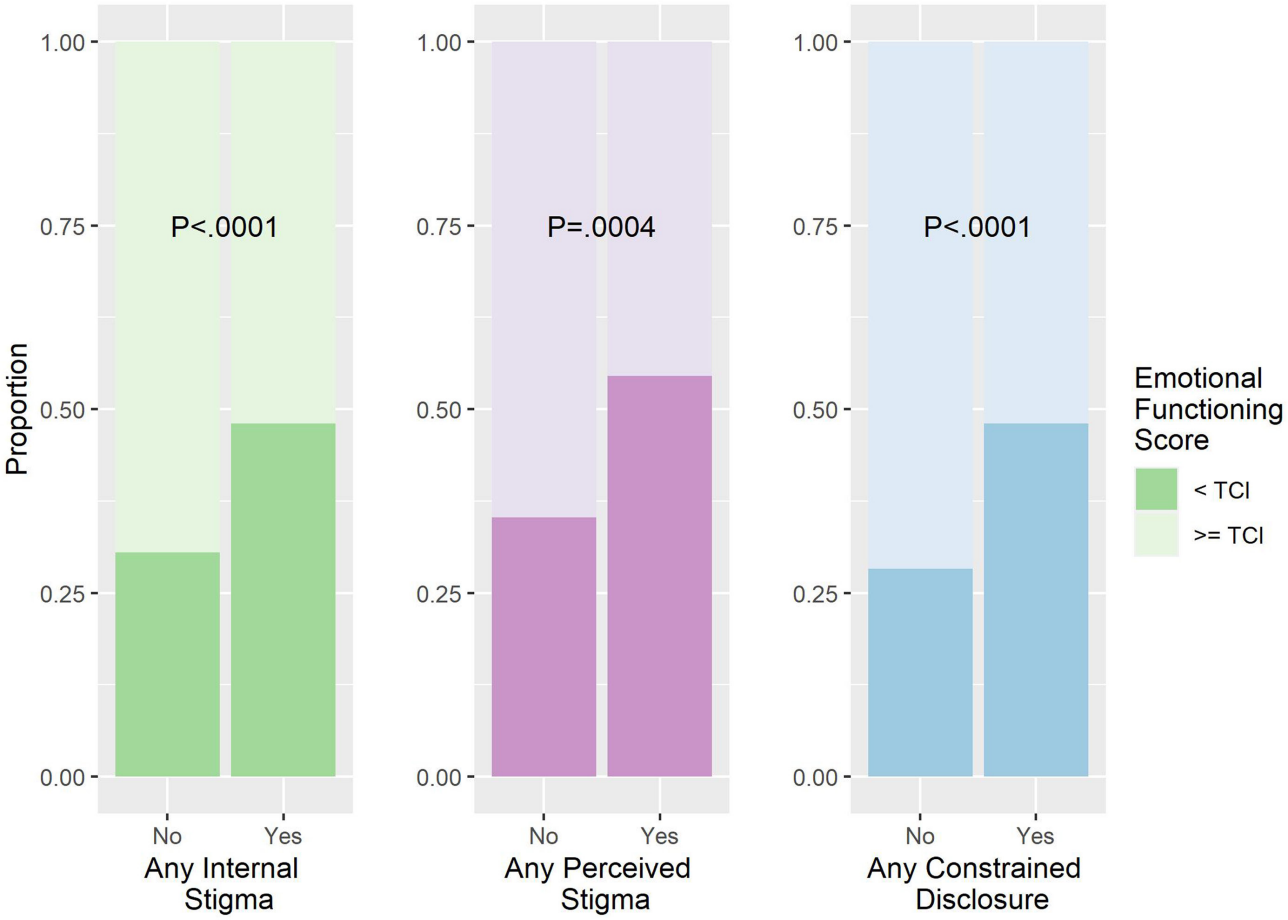
All types of stigma are associated with decreases in emotional functioning. TCI, threshold of clinical importance using EORTC QLQ‐C30 Emotional Functioning Scale.

Increased stigma was associated with a decrease in emotional functioning as measured by the Emotional Functioning (EF) Scale of the QLQ‐C30 (Figure 1). Participants who reported any level of internalized stigma were more likely to score below the threshold for clinical importance (TCI) (48% of participants with internalized stigma vs 31% without, *p* < 0.0001). Similarly, 55% of participants who reported any level of perceived stigma had an EF scale score below the TCI, versus 35% of participants who did not perceive stigma (*p* = 0.0004). Participants reporting any level of constrained disclosure also had lower scores (48% scored below the TCI, versus 28%, *p* < 0.0001). In general, participants reporting any level of any type of stigma had lower average emotional functioning scores than those who did not (Table S1). For all types of stigma, emotional functioning scores decreased monotonically with increasing levels of stigma on the 5‐level scale (Figure S1). Further analysis of the components of the EF scale demonstrated that no single emotional side effect was responsible for this effect. Depression, anxiety/worry, being tense, and irritability all contributed to decreased emotional function (Figure S2).

**FIGURE 2:**
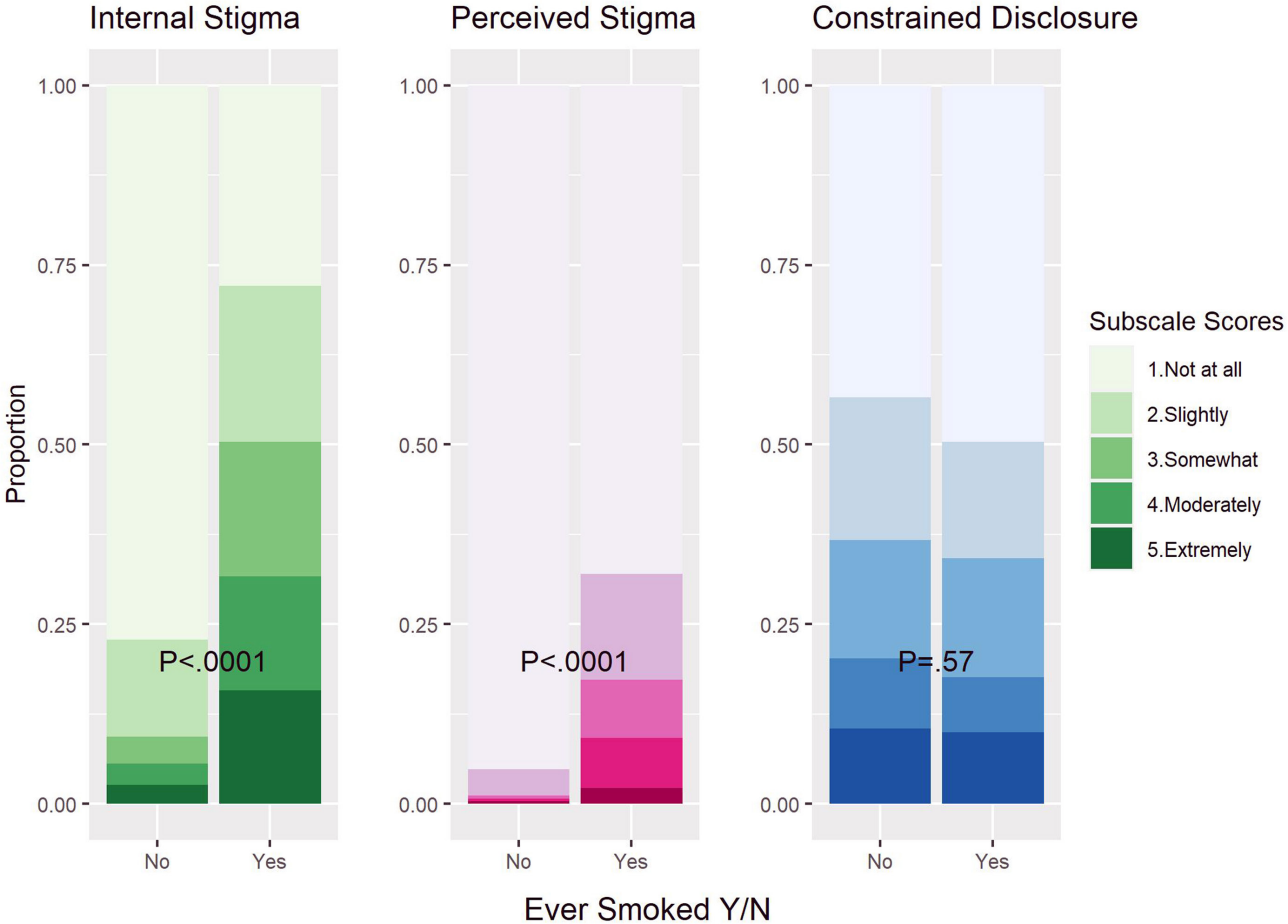
History of smoking predicts internalized stigma & perceived stigma but not constrained disclosure.

Participants who reported a history of current or former smoking experience higher levels of internalized stigma and perceived stigma (Figure 2, Table 2). Importantly, there was no association between reported smoking history and constrained disclosure. Seventy‐two percent of participants with a smoking history reported some level of internalized stigma, versus only 23% of those without a history of smoking (adjusted odds ratio 10.50, *p* < 0.0001). Similarly, 32% of participants with a smoking history reported any level of perceived stigma, versus 5% of those without a smoking history (odds ratio = 10.31, *p* < 0001). The association between a history of smoking and constrained disclosure was not significant, with an odds ratio of 0.84 and a *p*‐value of 0.34. Fifty percent of those with a smoking history reported constrained disclosure, versus 56% of never‐smokers. These models also revealed that after adjusting for smoking history, age over 70 was independently associated with a decreased likelihood of all types of stigma. Additionally, increasing time from initial survey submission was associated with decreasing likelihood of constrained disclosure but was not associated with any decrease in internalized or perceived stigma. Sensitivity analyses utilizing ordered logistic regression models with stigma as a 5‐level ordered variable yielded the same results with respect to stigma and smoking. Odds ratios of increasing levels of stigma were similarly strong for smoking history (*p* < 0.0001) for internalized and perceived stigma, and showed no relationship for constrained disclosure (*p* = 0.59). In the case of perceived stigma in particular, some cell sizes were very small for the participants without a smoking history, emphasizing the strong relationship between smoking and perceived stigma. Out of 267 participants with no smoking history, 254 reported “not at all” in response to the perceived stigma question, 10 reported “slightly,” and only one each reported experiencing the higher 3 levels of stigma.

**TABLE 2:** Associations between smoking history and stigma/constrained disclosure; multivariable model including age and time since initial survey submission.

Predictor	Internal stigma Yes (*N* = 257) vs No (*N* = 282)		Perceived Stigma Yes (*N* = 100) vs No (*N* = 439)		Constrained disclosure Yes (*N* = 288) vs No (*N* = 251)	
	OR (95% CI)	*p*‐Value	OR (95% CI)	*p*‐Value	OR (95% CI)	*p*‐Value
Smoking history (yes vs no)	10.50 (6.91, 15.94)	0.0001	10.31 (5.53, 19.20)	0.0001	0.85 (0.60, 1.21)	0.38
Age (<70 vs 70+)	0.44 (0.27, 0.72)	0.001	0.51 (0.28, 0.91)	0.02	0.61 (0.39, 0.95)	0.03
Time from initial submission (mos.)	0.99 (0.97, 1.00)	0.11	1.01 (0.99, 1.03)	0.25	0.98 (0.97, 1.00)	0.02
Gender (female vs male)	**Not included in model**		**Not included in model**		1.82 (1.19, 2.79)	0.01

*Note*: Odds ratio (OR) from logistic regression model with outcome stigma (yes/no).

Analyses revealed no difference between male and female participants in the likelihood of reporting internal or perceived stigma. Females were more likely to report constrained disclosure compared with males (57% vs 41%), and sex remained a significant covariate in multivariable analysis (*p* = 0.006). There were no significant differences in the reporting of any type of stigma between geographical regions.

**FIGURE 3:**
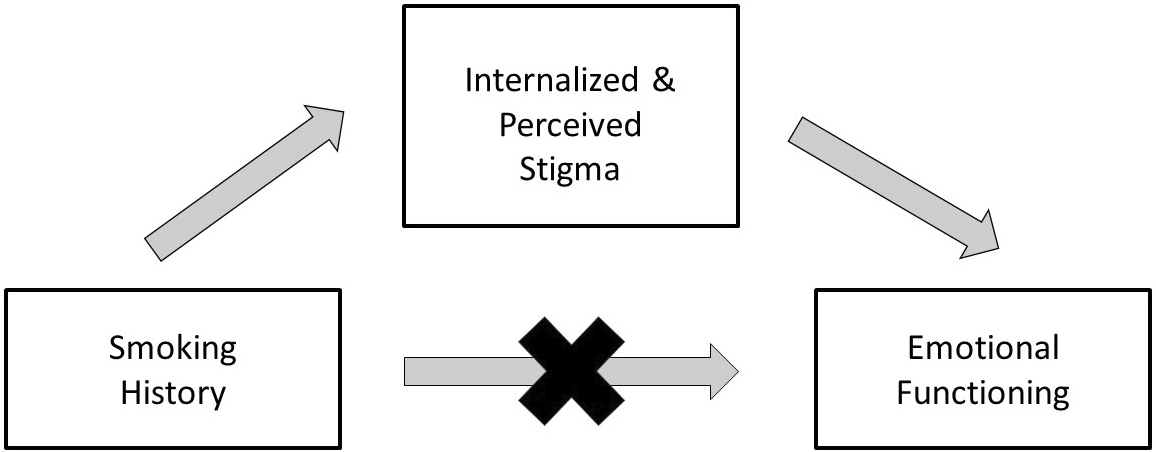
Internalized and perceived stigma, but not presence of a smoking history, predicts impairment in emotional functioning.

Because smoking history was associated with increased perceived and internalized stigma, and all types of stigma were associated with reduced emotional functioning, we asked if smoking history itself could predict lower emotional functioning. However, smoking status was not found to be associated with a reduction in emotional functioning score. These relationships are described in Figure 3. The distributions of scores were similar in participants with and without a smoking history (*p* = 0.18 by Wilcoxon signed‐rank test). An exploration of stigma as a mediator in a causal pathway between smoking and reduced emotional functioning scores was therefore not warranted.

## 4 DISCUSSION

### 4.1 The impact of stigma on emotional health

This study demonstrates the impact of utilizing an international registry to understand patient‐reported health‐related quality of life. This real‐world sample included over 500 complete cases of people diagnosed with lung cancer across a diverse geographical representation. Importantly, using the validated EORTC QLQ‐C30 Quality of Life subscale for emotional functioning, experiencing any type of lung cancer stigma (perceived stigma, internalized stigma, or constrained disclosure) was significantly associated with a decrease in Emotional Function. Prior literature has demonstrated that people with lung cancer have a greater level of psychosocial distress compared to other cancers^6^ and that stigma can impact quality of life, with a particular association with of depression.^23^, ^25^, ^40^, ^41^ In this analysis, multiple compounding emotional factors including anxiety, depression, irritability, and stress all contributed to the distress.

### 4.2 Smoking and lung cancer stigma

When examining smoking history, multiple trends that would be expected in the lung cancer population were evident within this real‐world sample. Presence of a smoking history was defined per the standard of greater than 100 cigarettes during one's lifetime.^42^ Older participants were more likely to have a smoking history, consistent with data on smoking patterns in the United States.^43^ Additionally, although numbers were small, people diagnosed with small cell lung cancer more often reported a smoking history than those with any stage of non‐small cell. People without a smoking history were more likely to be diagnosed with Stage IV disease, consistent with current lung cancer screening guidelines where those without a smoking history are not eligible for lung cancer screening by low‐dose computed tomography (LDCT).^44^

It has been known for decades that both people with and without smoking history can experience stigma that impacts their quality of life.^23^, ^25^ Consistent with a prior study,^31^ smoking history was significantly associated with levels of internal and perceived stigma. These findings are in agreement with common understanding of lung cancer risk factors—blaming oneself for developing the disease after participating in a high‐risk (albeit highly addictive) habit or having others believe the same. Experts have questioned the public health benefits of certain large‐scale anti‐tobacco campaigns in light of their potential for exacerbating stigma toward patients diagnosed with lung cancer.^45^ Interestingly, in the statistical models, age over 70 was also independently associated with a decreased likelihood of all types of stigma. This may be related to the timing of the Surgeon General's report^46^ and the concept that for most of their adult lives, smoking was normalized in this population and the anti‐tobacco campaigns were not prevalent.

Notably, even though a history of smoking was associated with internal and perceived stigma, and stigma was associated with poor emotional function, there was no direct association between smoking history and emotional function in this analysis. This indicates that the smoking history itself, which can be linked to a number of comorbidities, is not causative in producing the observed the emotional distress. The impact of the guilt and blame of lung cancer stigma is the primary driver on the emotional impacts.

### 4.3 Constrained disclosure of lung cancer

More than half of all participants in this study experienced constrained disclosure—avoidance of telling others about their lung cancer diagnosis. There are many potential reasons that one would not reveal a cancer diagnosis. A prior study of men with prostate cancer implicated men's low perceived need for support, fear of stigmatization, minimization to aid with coping, fear of workplace repercussions, and the desire to avoid burdening others.^47^ While we did not assess the reasons for the lack of disclosure in this study, we know that for lung cancer, stigma remains a significant factor in coping with and treating the disease, as detailed in the introduction.

Interestingly, constrained disclosure was not associated with presence of a smoking history. More than half of the people with no smoking history avoided telling others about their lung cancer diagnosis. Moreover, this constrained disclosure demonstrated similar significant association with reduced emotional functioning (anxiety, depression, etc.) as the other types of stigma. Although there was previous understanding that people without a smoking history experience lung cancer stigma,^23^ these findings help elucidate the specific subtype of stigma experienced by many. They are consistent with one prior site‐based study that also found no relationships between constrained disclosure and smoking history.^31^ This has important implications for lung cancer survivorship. There is an increasing rate of diagnoses of lung cancer in those without a smoking history, particularly in young women.^48^, ^49^ Our findings suggest that this population is still highly susceptible to the constrained disclosure subtype of stigma.

### 4.4 Clinical implications

These results coupled with other literature demonstrate that there needs to be clear recognition of the impact of lung cancer stigma on emotional functioning of people diagnosed with lung cancer. One study reported that 98% of patients with lung cancer reported stigma‐related consequences including emotional impacts, differences on treating, loss of hope, and negative impacts on treatment.^8^ Clinicians need to be sensitive to the fact that, regardless of smoking status, people with lung cancer may experience stigma and that feeling stigmatized can affect treatment adherence and may require referral for additional support services.

Interventions are currently being studied to reduce the long‐term impact of lung cancer stigma and improve emotional wellbeing. Examples include empathic communication training for providers^50^, ^51^ and acceptance and commitment therapy for those who are experiencing internalized stigma.^52^

As discussed above, constrained disclosure impacted people with lung cancer even without any smoking history. A recent study has shown that high levels of self‐compassion can mitigate internalized stigma but not constrained disclosure.^53^ Taken together, there are significant implications for psychosocial care of people with diagnosed with lung cancer as the constrained disclosure is negatively impacting wellbeing but may not be responsive to compassion‐based interventions. Provider‐focused or other types of interventions may be required to address the impact of stigma in the growing population of people with lung cancer unrelated to smoking history.

### 4.5 Limitations & future considerations

As with all research, this study has limitations. The Lung Cancer Registry is only available online at this time, which biases the data to those collected from people with access to technology and the internet. The sample is also overrepresented in both women and those of higher educational status. Another potential limitation is that the smoking history data is entirely self‐reported. People may not be reliable historians when reporting their own tobacco use.^54^

Our findings add real‐world evidence to the body of literature that all types of lung cancer stigma are strongly associated with decreased emotional functioning and that the presence of stigma results in a significant impact on health‐related quality of life for people with lung cancer. Interestingly, even those diagnosed with lung cancer without a smoking history experience stigma—specifically constrained disclosure. Hamann et al.^55^ have described the need for multilevel, coordinated, interdisciplinary interventions across the entire lung cancer continuum. These multi‐level interventions will need to address all subtypes of stigma, and more research is needed to understand how effective potential interventions may be for people experiencing different subtypes.

## AUTHOR CONTRIBUTIONS

**Kari Chansky:** Data curation (lead); formal analysis (lead); investigation (equal); methodology (lead); writing – original draft (equal); writing – review and editing (equal). **Maureen Rigney:** Conceptualization (equal); investigation (equal); writing – original draft (equal); writing – review and editing (equal). **Jennifer C. King:** Conceptualization (equal); funding acquisition (lead); investigation (equal); supervision (lead); writing – original draft (equal); writing – review and editing (equal).

## FUNDING INFORMATION

Funding for this work was provided by GO2 for Lung Cancer through generous support of the Lung Cancer Registry by Amgen, Bristol Myers Squibb, Foundation Medicine, Genentech, Lung Ambition Alliance, Mirati, Novartis, and Takeda.

## CONFLICT OF INTEREST STATEMENT

JCK reports consulting and advisory board participation, all paid to GO2 for Lung Cancer, from Amgen, Boehringer Ingelheim, Bristol Myers Squibb, and EQRX. MR reports consulting and advisor board participation, all paid to GO2 for Lung Cancer, from Amgen, AstraZeneca, Jazz, Novartis Global, and Gilead.

## ETHICS STATEMENT

All ethics guidelines were followed during the conduct of this study.

## PATIENT CONSENT STATEMENT

All participants in this study provided informed consent under an IRB‐approved protocol.

## Supporting information


Figure S1.

Figure S2.

Table S1.

Data S1.

## Data Availability

Data are available by request from the authors or via email to registry@go2.org. The data that support the findings of this study are available from the corresponding author upon reasonable request.
