# Supplementary figures and images for: Real‐world analysis of the relationships between smoking, lung cancer stigma, and emotional functioning
Source: Cancer Med. 2024 Jan 12;13(3):e6702. doi: 10.1002/cam4.6702 (PMC10905230; doi:10.1002/cam4.6702)

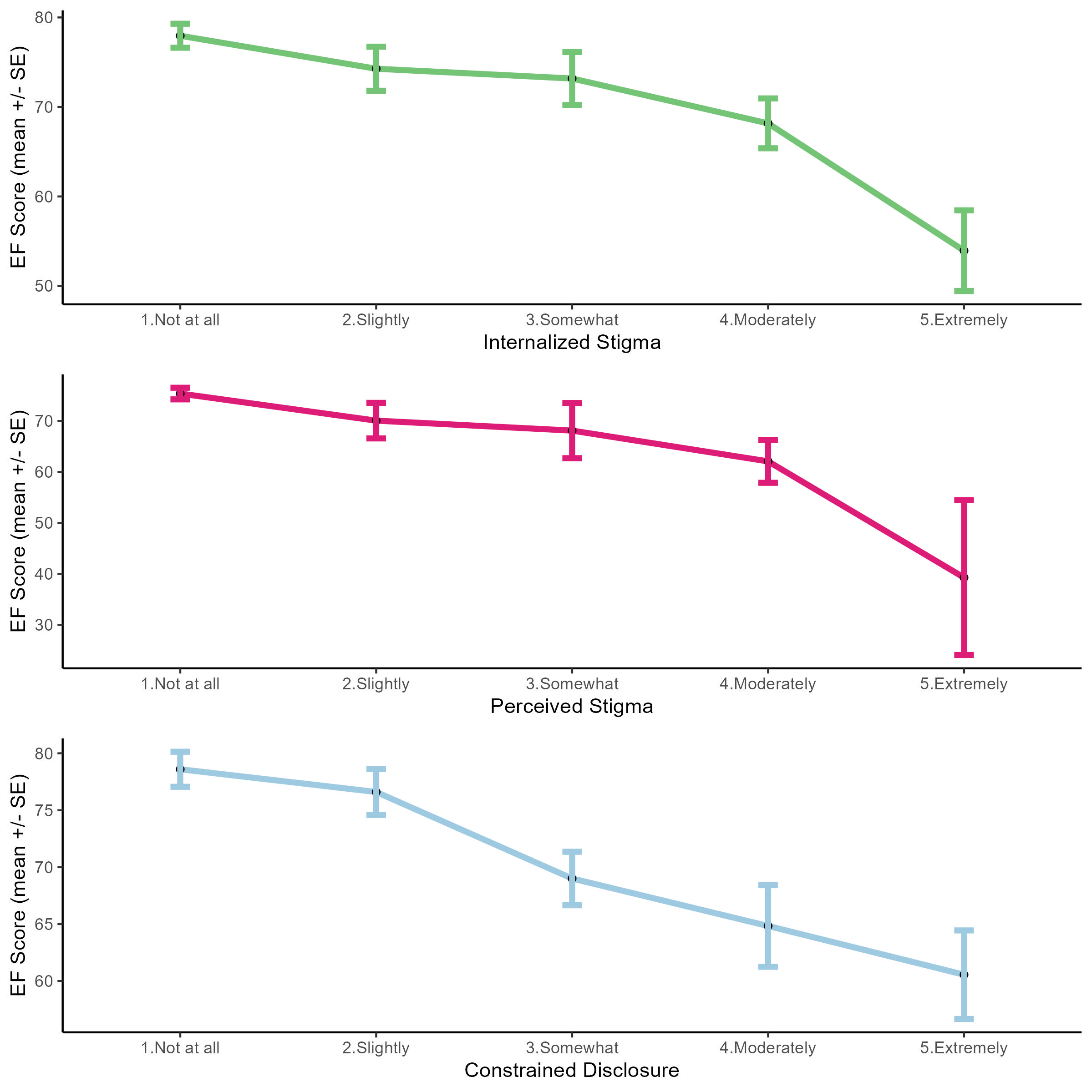

Supplement: Supplementary file 1 — Figure S1. [file CAM4-13-e6702-s002.jpg]

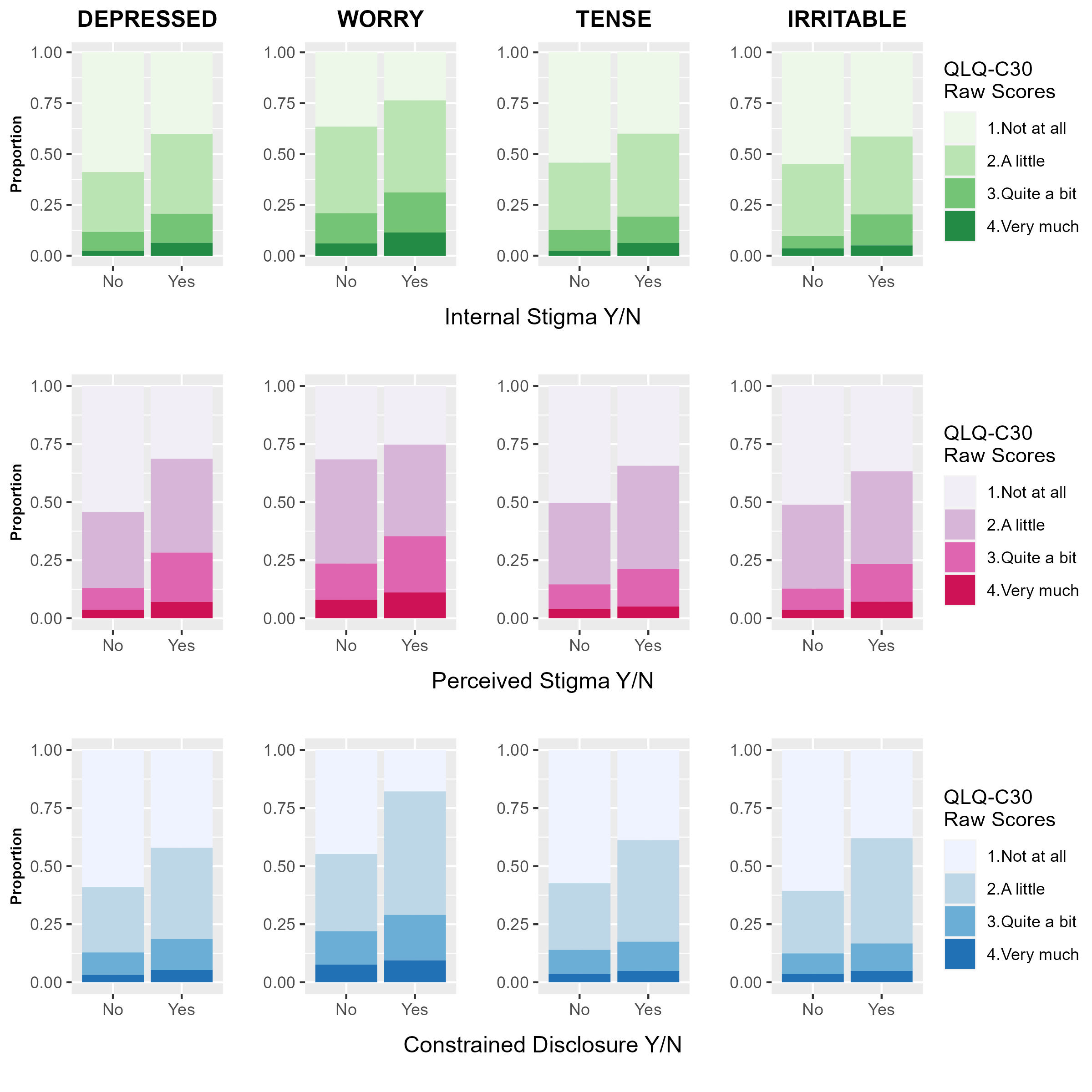

Supplement: Supplementary file 2 — Figure S2. [file CAM4-13-e6702-s003.jpg]
