# Supplementary material for: Real‐world analysis of the relationships between smoking, lung cancer stigma, and emotional functioning
Source: Cancer Med. 2024 Jan 12;13(3):e6702. doi: 10.1002/cam4.6702 (PMC10905230; doi:10.1002/cam4.6702)
Supplement: Supplementary file 3 — Table S1. [file CAM4-13-e6702-s004.docx]

Supplemental Table 1. Mean EORTC QLC-C30 Emotional Functioning Score for each type of stigma experienced.

|  | **Mean Emotional Functioning Score (95% C.I.)** | |
| --- | --- | --- |
| **Type of Stigma** | **Experienced Stigma** | **Did Not Experience** |
| Internalized Stigma | 68.8 (65.7 - 72.0) | 78.0 (75.3 - 80.6) |
| Perceived Stigma | 65.8 (60.6 - 71.0) | 75.4 (73.1 - 77.6) |
| Constrained Disclosure | 69.3 (66.5 - 72.0) | 78.6 (75.6 - 81.6) |
