# Supplementary material for: Real‐world analysis of the relationships between smoking, lung cancer stigma, and emotional functioning
Source: Cancer Med. 2024 Jan 12;13(3):e6702. doi: 10.1002/cam4.6702 (PMC10905230; doi:10.1002/cam4.6702)
Supplement: Supplementary file 4 — Data S1. [file CAM4-13-e6702-s001.docx]

**Supplemental Figure 1**. Mean scores from the EORTC QLQ-C30 Emotional Functioning Scale increase with increasing reported levels of stigma.

**Supplemental Figure 2**. Individual component scores of the Emotional Functioning Scale are all impacted by increasing levels of stigma.
